# An Approach to Paracyclophane-Based Tetrathiafulvalenes: Synthesis and Characterization of a *Pseudo-Geminal* [2.2]Paracyclophane 1,3-Dithia-2-Thione

**DOI:** 10.3390/molecules25225262

**Published:** 2020-11-11

**Authors:** Lucian G. Bahrin, Henning Hopf, Peter G. Jones, Mihail L. Birsa, Laura G. Sarbu

**Affiliations:** 1 Petru Poni Institute of Macromolecular Chemistry, Intelcenter. 41A Grigore Ghica Vodă Alley, 700487 Iasi, Romania; bahrin.lucian@icmpp.ro; 2 Institute of Organic Chemistry, Technical University of Braunschweig, Hagenring 30, D-38106 Braunschweig, Germany; h.hopf@tu-bs.de; 3 Institute of Inorganic and Analytical Chemistry, Technical University of Braunschweig, Hagenring 30, D-38106 Braunschweig, Germany; p.jones@tu-bs.de; 4 Department of Chemistry, Alexandru Ioan Cuza University of Iasi, 11 Carol I Blvd., 700506 Iasi, Romania

**Keywords:** [2.2]paracyclophanes, dithiocarbamates, 1,3-dithiolium salts, trithiones

## Abstract

The synthesis of paracyclophane-based tetrathiafulvalene precursors is described in the context of the importance of these compounds in the field of material chemistry. *Pseudo-geminal* bis(1,3-dithia-2-thione) was synthesized via the corresponding 1,3-dithiol-2-ylium salt. The latter was obtained by a synthetic procedure that involves 4,15-bis(acetyl)[2.2]paracyclophane, a new compound of interest for many researchers.

## 1. Introduction

[2.2]Paracyclophane derivatives have been the subject of particular interest since their first appearance in the literature, more than seven decades ago [1,2,3]. Since then, most studies have been devoted to the elucidation of the structural characteristics of [2.2]paracyclophanes, particularly their geometry and steric properties, transannular interactions, and ring strain [4,5,6]. Most of the unique properties of these cyclophanes are the result of the rigid framework and the short distance between the two aromatic rings within the [2.2]paracyclophane unit. In one such application, unsaturated cyclophane bis(esters) provided the corresponding ladderanes by intramolecular photocyclization [7]. The [2.2]paracyclophane core can undergo chemical transformations specific to both aliphatic and aromatic compounds, resulting in a wide variety of functionalized [2.2]paracyclophanes. Both the parent hydrocarbon and its derivatives have been used in asymmetric catalysis [8,9,10,11], optoelectronics [12], and polymer synthesis [13].

Special attention has been paid to the ability of these compounds to form charge transfer complexes [14]. Tetrathiafulvalene (TTF) and its derivatives have been extensively studied with respect to their applications as organic metals and superconductors [15,16]. These properties are a consequence of the π-donor properties of TTF and of its important intermolecular interactions in the solid state through extended π-orbitals. The design of new tetrathiafulvalene derivatives has targeted those systems where the intermolecular interactions between planar molecules are more efficient and the solid-state architecture tends to organize as stacks or layers, with their long axes mutually parallel [17].

We report here the synthesis and characterization of *pseudo-geminal* [2.2]paracyclophane 1,3-dithia-2-thione as a precursor for hybrid [2.2]paracyclophane-tetrathiafulvalene systems.

## 2. Results and Discussion

**Scheme 1:**
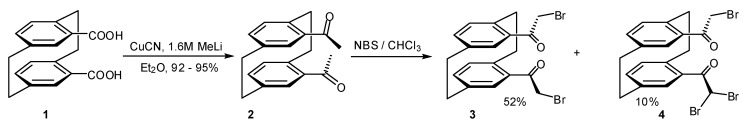
Synthesis of 4,15-bis(acetyl)[2.2]paracyclophane and its bromination products.

**Scheme 2:**
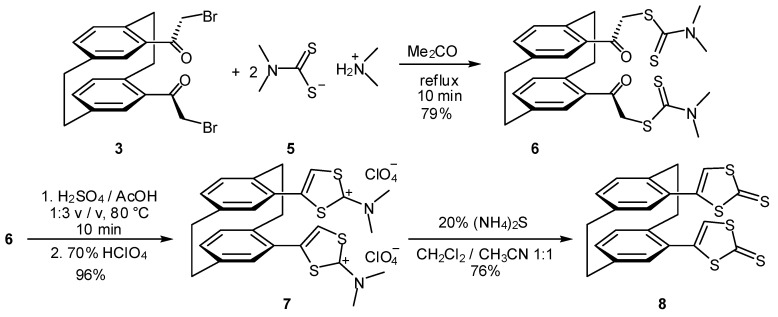
Synthesis of *pseudo-geminal* [2.2]paracyclophane trithiones.

The synthetic pathway for the synthesis of *pseudo-geminal* [2.2]paracyclophane 1,3-dithia-2-thione is depicted in Scheme 1 and Scheme 2. The reactions use 4,15-bis(carboxyl)[2.2]paracyclophane (**1**) as the starting material. 

**Figure 1:**
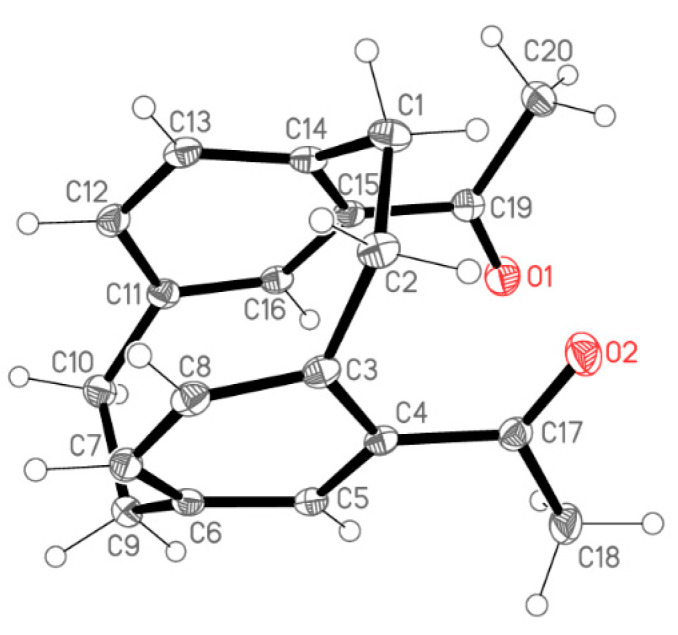
Molecular structure of 4,15-bis(acetyl)[2.2]paracyclophane **2**; ellipsoids represent 30% probability levels [19]. O1 and O2 represent the two oxygen atoms from 4,15-bisacetyl[2.2]paracyclophane.

**Figure 2:**
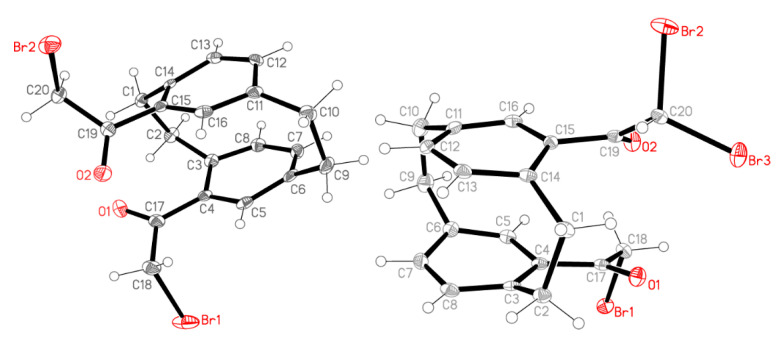
Molecular structure of 4,15-bis(bromoacetyl)[2.2]paracyclophane (**3**) (**left**) and 4-bromoacetyl-15-dibromoacetyl[2.2]paracyclophane (**4**) (**right**); ellipsoids represent 50% probability levels [20]. O1 and O2 represent the two oxygen atoms and Br1, Br2 and Br3 the bromine atoms.

As can be seen in Scheme 1, *pseudo-geminal* derivative **1** [18] was converted to the *pseudo-geminal* bis(acetyl) derivative **2** by treatment with methyl lithium in the presence of copper(I) cyanide. We note that 4,15-bis(acetyl)[2.2]paracyclophane is a new derivative that has great potential to become an important synthetic intermediate in [2.2]paracyclophane chemistry. After the reaction workup, the desired product was isolated in 93% yield. Single crystals of **2** were obtained by layering hexane over a solution of **2** in dichloromethane; the structure of **2** is shown in Figure 1. Several bromination methods of **2** were investigated, involving molecular bromine, copper(I) bromide, and *N*-bromosuccinimide as brominating agents. Among these, *N*-bromosuccinimide proved to be the most efficient, providing the corresponding bis(dibromide) **3** in reasonable yield (52%) as well as the tribrominated derivative **4** as a side-product (10% yield). Single crystals of both **3** and **4** have been obtained by the same method and the structures are shown in Figure 2.

The next step involved the synthesis of bisdithiocarbamate derivative **6** is by treatment of **3** with dimethylammonium *N*,*N*-dimethyldithiocarbamate **5**, as presented in Scheme 2. The reaction proceeds readily in refluxing acetone, providing the desired product in 79% yield. From a series of various aminocarbodithioates derived from secondary amines (pyrrolidine, piperidine, morfoline), the use of the dimethylammonium derivative provided the best yield and a cleaner crude reaction product. Dithiocarbamate **6** was then converted into bis(1,3-dithiolium) perchlorate **7** through a method extensively used by us in the past [21,22,23,24], which involved heating **6** in a mixture of sulfuric and acetic acid over a period of 10 min, followed by addition of perchloric acid to the reaction mixture. Bis(1,3-dithiolium) perchlorate **7** was thereby obtained in 96% yield.

The cyclization of dithiocarbamates **6** was accompanied by important spectral changes. The IR spectra revealed the disappearance of the absorption band corresponding to the carbonyl group (1676 cm^−1^) and the presence of new, strong, and broad absorption bands at 1100–1200 cm^−1^, corresponding to the perchlorate anion. Heterocyclization of dithiocarbamates **6** is also supported by the NMR spectrometry. Thus, the ^1^H-NMR spectrum of the 1,3-dithiol-2-ylium perchlorate indicates the disappearance of the signal of the α-carbonyl hydrogen atom from compound **6** (4.08 ppm). The ^13^C-NMR spectrum also supports the synthesis of 1,3-dithiolium salt **7** by the disappearance of the carbonyl and thiocarbonyl carbon atoms and the appearance of a new signal at very low field (186 ppm) which corresponds to the electron-deficient C-2 atom (see Supplementary Materials). Finally, the desired bis(1,3-dithia-2-thione) **8** was obtained by treatment of **7** with ammonium sulfide. Although sodium sulfide was also investigated as a sulfur nucleophile, ammonium sulfide provided the best yield (76%) and a better quality of the crude reaction mixture. NMR spectra support the formation of compound 8 by the disappearance of the signals of the dimethylamino groups in ^1^H NMR. The ^13^C-NMR spectrum also indicates the disappearance of the signals of the methyl carbon atoms and that of the electron-deficient C-2 atom. The formation of the carbon–sulfur double bond is accompanied by the appearance of a new signal at 211.1 ppm (see Supplementary Materials). 

**Figure 3:**
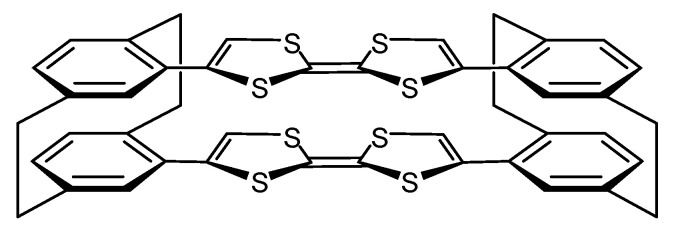
A possible stereoisomer of *pseudo-geminal* [2.2]paracyclophane-based tetrathiafulvalene.

1,3-Dithia-2-thiones derivatives are important precursors for the corresponding substituted tetrathiafulvalenes either by a homocoupling or heterocoupling approach. Under homocoupling conditions, *pseudo-geminal* derivative **8** should provide paracyclophane-based tetrathiafulvalenes, one of the possible stereoisomers being depicted in Figure 3. Preliminary investigations on phosphite-mediated homocoupling of **8** have not provided convincing results so far. These studies are still under evaluation.

## 3. Materials and Methods

### 3.1. Chemistry

Melting points were obtained on a *KSPI* melting-point meter (A. KRÜSS Optronic, Hamburg, Germany) and are uncorrected. IR spectra were recorded on a Bruker Tensor 27 instrument (Bruker Optik GmbH, Ettlingen, Germany). NMR spectra were recorded on a Bruker 500 MHz spectrometer (Bruker BioSpin, Rheinstetten, Germany). Chemical shifts are reported in ppm downfield from TMS. Mass spectra were recorded on a Thermo Scientific ISQ LT instrument (Thermo Fisher Scientific Inc., Waltham, MA, USA). All reagents were commercially available and used without further purification.

#### 3.1.1. 4,15-Bis(acetyl)[2.2]paracyclophane (**2**)

MeLi (1.6 M in Et_2_O, 12.5 mL, 20 mmol) was added dropwise to a suspension of CuCN (0.9 g, 10 mmol) in Et_2_O (20 mL). After 5 min, 4,15-bis(carboxyl)[2.2]paracyclophane **1** (0.296 g, 1 mmol) was added and the reaction mixture was left at 0 °C for 20 min. A solution of NH_4_Cl was then added and the organic layer was extracted with CH_2_Cl_2_ and dried over Na_2_SO_4_. Evaporation and recrystallization from ethanol gave **2** (0.272 g, 93%) as colorless crystals. m.p. 145–146 °C. IR (ATR, cm^−1^) 2927, 2851, 1666, 1585, 1552, 1434, 1341, 1261, 1203, 958, 850, 729, 636, 606, 576. ^1^H-NMR (CDCl_3_) δ 6.85–6.89 (m, 2H), 6.54–6.58 (m, 4H), 3.79–3.82 (m, 2H), 3.1 (s, 4H), 2.97–2.99 (m, 2H), 2.35 (s, 6H). ^13^C-NMR (CDCl_3_) δ 200.3, 140.9, 139.4, 138.4, 135.90, 135.88, 132.7, 35.2, 34.8, 29.3. MS (EI) *m/z*: 292.2 (M^+^, 70%) for C_20_H_20_O_2_.

#### 3.1.2. Bromination of 4,15-bis(acetyl)[2.2]paracyclophane

NBS (1.2 g, 6.7 mmol) and *p*-TsOH (0.25 g, 1.34 mmol) were added to a solution of **2** (0.978 g, 3.35 mmol) in CHCl_3_ (30 mL). The reaction mixture was refluxed for 30 min and then cooled to rt. Subsequent washing with water and sodium bicarbonate solution (5%) provided the crude reaction mixture, which was purified by column chromatography on silica gel using dichloromethane/pentane 1:1 as the eluent. Compounds **3** and **4** were isolated following this procedure.

#### 3.1.3. 4,15-Bis(bromoacetyl)[2.2]paracyclophane (**3**)

0.78 g 52%, yellow crystals. m.p. 132–133 °C. IR (ATR, cm^−1^) 3040, 2957, 2931, 2852, 2091, 1665, 1589, 1550, 1480, 1426, 1008, 965, 699, 629, 608. ^1^H-NMR (CDCl_3_) δ 6.89 (d, ^4^*J* = 2.3 Hz, 2H), 6.72 (dd, ^3^*J* = 8.1, ^4^*J* = 2.3 Hz, 2H), 6.65 (d, ^3^*J* = 8.1 Hz, 2H), 4.38 and 4.27 (ABq, ^2^*J* = 12.4 Hz, 4H), 3.73–3.81 (m, 2H), 3.05–3.13 (m, 6H). ^13^C-NMR (CDCl_3_) δ 194.3, 141.1, 139.8, 136.8, 136.2, 135.1, 132.8, 35.4, 34.7, 34.1. MS (EI) *m/z*: 448 (M^+^, 8%) for C_20_H_18_^79^Br_2_O_2_.

#### 3.1.4. 4-Bromoacetyl-15-(dibromo)acetyl[2.2]paracyclophane (**4**)

0.1 g, 10%, yellow crystals. m.p. 156–157 °C. IR (ATR, cm^−1^) 2941, 2874, 2046, 1657, 1578, 1540, 1470, 1436, 1011, 685, 621. ^1^H-NMR (CDCl_3_) δ 6.93–6.98 (m, 1H), 6.87–6.92 (m, 1H), 6.74–6.81 (m, 2H), 6.64–6.73 (m, 2H), 6.48 (s, 1H), 4.49 and 4.33 (ABq, ^2^*J* = 12.5 Hz, 2H), 3.93–4.01 (m, 1H), 3.49–3.58 (m, 1H), 3.09–3.25 (m, 5H), 3.0–3.09 (m, 1H). ^13^C-NMR (CDCl_3_) δ 193.2, 189.8, 141.6, 140.9, 140.2, 139.8, 137.5, 136.7, 136.6, 136.1, 134.3, 133.4, 132.8, 132.5, 42.6, 36.4, 35.1, 34.8, 34.7, 33.6. MS (EI) *m/z*: 529.8 (M^+^, 20%) for C_20_H_17_^79^Br_3_O_2_.

#### 3.1.5. 4,15-Bis(*N*,*N*-dimethyldithiocarbamate) (**6**)

A solution of *N*,*N*-dimethyldithiocarbamate **5** (0.9 g, 3.58 mmol) in acetone/water (1:1, 8 mL) was added to a solution of **3** (0.805 g, 1.79 mmol) in acetone (20 mL). The reaction mixture was refluxed for 30 min, then cooled and poured into water. The resulting solid was filtered and purified by recrystallization from ethanol to give 0.75 g, 79%, colorless crystals. m.p. 197–198 °C. IR (ATR, cm^−1^) 2962, 2787, 2165, 2026, 1676, 1499, 1376, 1290, 1246, 1144, 972, 857, 740, 723, 645, 525, 506. ^1^H-NMR (CDCl_3_) δ 7.05–7.08 (m, 2H), 6.70–6.74 (m, 2H), 6.62–6.66 (m, 2H), 4.08 (s, 4H), 3.83–3.90 (m, 2H), 3.06 (s, 6H), 3.12 (s, 6H), 3.12–3.19 (m, 4H), 3.04–3.09 (m, 2H). ^13^C-NMR (CDCl_3_) δ 196.1, 195.7, 140.9, 139.6, 137.2, 136.3, 136.1, 132.5, 47.5, 45.7, 41.7, 35.1, 34.8. MS (EI) *m/z*: 530.0 (M^+^, 3%) for C_26_H_30_N_2_O_2_S_4_.

#### 3.1.6. 4,15-Bis(1,3-dithiol-2-ylium) perchlorate (**7**)

Dithiocarbamate **6** (0.7 g, 1.3 mmol) was added to a mixture of sulfuric acid (1 mL) and acetic acid (3 mL), and the resulting solution was heated to 80 °C for 10 min. The reaction mixture was then left to cool to room temperature and HClO_4_ 70% (0.5 mL) was added. The resulting precipitate was then filtered off, washed thoroughly with water, and recrystallized from ethanol, yielding the desired 1,3-dithiolium perchlorate **7** in the form of colorless crystals (0.885 mg, 96%). m.p. 305–306 °C. IR (ATR, cm^−1^) 1598, 1441, 1080, 802, 787, 724, 622, 566. ^1^H-NMR (DMSO-*d*6) δ 7.63 (s, 2H), 6.80–6.93 (m, 6H), 3.51–3.59 (m, 12H), 3.46–3.51 (m, 2H), 3.28–3.34 (m, 2H), 3.13–3.20 (m, 2H), 3.07–3.13 (m, 2H). ^13^C-NMR (DMSO-*d*6) δ 186.6, 141.4, 138.5, 137.4, 137.3, 135.6, 131.6, 129.1, 120.9, 48.2, 47.2, 34.3, 34.2. MS (EI) *m/z*: 496.1 (M^+^-ClO_4,_ 8%) for C_26_H_30_N_2_S_4_]^+^.

#### 3.1.7. 4,15-Bis(1,3-dithia-2-thione) (**8**)

(NH_4_)_2_S (20%, 0.56 mL, 0.6 mmol) was added to a solution of 1,3-dithiolium perchlorate **7** (0.17 g, 0.25 mmol) in a mixture of CH_2_Cl_2_ and CH_3_CN (1:1, 10 mL). The reaction mixture was stirred at room temperature for 5 h and then poured into water. Extraction with CH_2_Cl_2_, evaporation, and purification from ethanol provided bis(trithione) **8** as a yellow solid (0.358 g, 76%). m.p. 242–243 °C. IR (ATR, cm^−1^) 1587, 1453, 1403, 1192, 1070, 1054, 876, 789, 712, 513. ^1^H-NMR (CDCl_3_) δ 6.94 (s, 2H), 6.72–6.75 (m, 2H), 6.68–6.72 (m, 2H), 6.54–6.58 (m, 2H), 3.63–3.72 (m, 2H), 3.15–3.24 (m, 4H), 3.04–3.11 (m, 2H). ^13^C-NMR (CDCl_3_) δ 211.1, 144.9, 140.6, 136.0, 136.5, 134.1, 131.3, 130.6, 124.8, 34.8, 34.6. MS (EI) *m/z*: 471.9 (M^+^, 94%) for C_22_H_16_S_6_.

### 3.2. X-ray Structure Determination

Crystals were mounted in inert oil on glass fibers and transferred to the cold gas stream of an Oxford Diffraction diffractometer (Oxford Diffraction Limited, Abingdon, UK) (**2**: Nova A using mirror-focussed Cu *K*α radiation; **3** and **4**: Xcalibur E using monochromated Mo *K*α radiation). Absorption corrections were implemented on the basis of multi-scans. The structures were refined anisotropically on *F*^2^ using the programs SHELXL-1997 [25] (2) or -2018 [26] (**3** and **4**). Due to the inherent strain of cyclophane systems, hydrogen atoms of the cyclophane rings were refined freely but with C–H distance restraints; other hydrogens were included using rigid methyl groups or a riding model starting from calculated positions (see Supplementary Materials).

Special features: Structure **3** was refined as a pseudo-merohedral twin based on a pseudo-orthorhombic cell generated by the matrix –1 0 0/1 0 2/0 1 0. The TWIN matrix was 1 0 0/–1 0 0/–1 0 –1, and the scale factor (relative volume of the smaller twin component) was refined to 0.0791(8). For structure **4**, the largest difference peaks (1.3 and 1.0 e/A^3^) may correspond to an alternative position for the entire molecule (e.g., with the CHBr_2_ and CH_2_Br groups exchanged) or to contamination by a more highly brominated species. However, attempts to refine the peaks as alternative bromine positions led to occupation factors of only ca. 1%.

## 4. Conclusions

The synthesis of paracyclophane-based tetrathiafulvalenes precursors is described in the context of the importance of these compounds in materials chemistry. *Pseudo-geminal* bis(1,3-dithia-2-thione) was synthesized via the corresponding 1,3-dithiol-2-ylium perchlorate. The latter was obtained by a synthetic procedure that involves 4,15-bisacetyl[2.2]paracyclophane, a new derivative that opens up a new range of possibilities in [2.2]paracyclophane chemistry. We hope to report on the conversion of **8** into its TTF-derivative in the near future.

## Supplementary Materials

The following are available online. Crystallographic data. Table S1: Crystallographic data and structure refinement details for compounds **2**–**4**. Table S2: Elemental analysis data for compounds **2**–**4** and **6**–**8**. Copies of ^13^C-NMR spectra.
