# Supplementary material for: An Approach to Paracyclophane-Based Tetrathiafulvalenes: Synthesis and Characterization of a Pseudo-Geminal [2.2]Paracyclophane 1,3-Dithia-2-Thione
Source: Molecules. 2020 Nov 11;25(22):5262. doi: 10.3390/molecules25225262 (PMC7698277; doi:10.3390/molecules25225262)
Supplement: Supplementary file 1 [file molecules-25-05262-s001.pdf]

An approach to paracyclophane-based tetrathiafulvalenes: Synthesis and characterization of a *pseudo-geminal* [2.2]paracyclophane 1,3-dithia-2-thione

Lucian G. Bahrin[1,2], Henning Hopf,[2] Peter G. Jones,[3] M. Lucian Birsa [2,4,*] and Laura G. Sarbu [2,4,*]

Supplementary Material

1. Crystallographic data for compounds 2-4 **S2**

2. Elemental analysis **S3**

3. Copies of ^{13}C NMR spectra **S4-S9**

1. Crystallographic data for compounds 2–4

Table 1. Crystallographic data and structure refinement details for compounds **2**–**4**.

Compound	2	3	4
Formula	$C_{20}H_{20}O_2$	$C_{20}H_{18}Br_2O_2$	$C_{20}H_{17}Br_3O_2$
M_r	292.36	450.16	529.07
Cryst. size (mm)	0.25 x 0.2 x 0.02	0.25 x 0.1 x 0.06	0.2 x 0.2 x 0.1
Crystal system	orthorhombic	monoclinic	orthorhombic
Space group	*Pbca*	$P2_1/n$	*Pbca*
Temperature (°C)	-172	-172	-173
a (Å)	15.2888(4)	9.1716(4)	15.1453(5)
b (Å)	11.3520(4)	11.6326(3)	11.7710(4)
c (Å)	16.8381(6)	16.8147(5)	20.3761(6)
α (°)	90	90	90
β (°)	90	105.306(4)	90
γ (°)	90	90	90
V (Å^3)	2922.4	1730.31	3632.5
Z	8	4	8
D_x (Mg m^{-3})	1.329	1.728	1.935
λ (Å)	1.54184	0.71073	0.71073
μ (mm^{-1})	0.66	4.7	6.8
Transmissions	0.840 – 1.000	0.649 – 1.000	0.712 – 1.000
F(000)	1248	896	2064
$2\theta_{max}$	152.4	52.7	56.6
Refl. measured	30270	76075	73369
Refl. indep.	3052	3640	4505
R_{int}	0.057	0.056	0.074
Parameters	225	242	250
Restraints	15	15	15
wR(F^2, all refl.)	0.098	0.102	0.061
R(F, >4σ(F))	0.037	0.039	0.029
S	1.03	1.08	1.03
Max. Δ*p* (e Å^{-3})	0.22, –0.20	1.75, –1.17	1.28, –0.46

All three structures show the usual features corresponding to the strain of cyclophane ring systems, e.g., lengthened single bonds and increased angles in the bridges, flattened boat shapes for the rings, and decreased ring angles at the bridgehead atoms. Compound **2** displays opposite orientations of the two acetyl groups towards the ethano bridges of the [2.2]paracyclophane core, *cf.* torsion angles C3—C4—C17—O2 –14.1(2), C14—C15—C19—O1 154.6(1)°. Compound **3** is similarly configured [C3—C4—C17—O1 –9.6(7), C14—C15—C19—O2 134.3(5)°], and its bromomethylene groups display similar torsion angles [C4—C17—C18—Br1 –73.3(5), C15—C19—C20—Br2 –81.1(4)°]; the molecular packing involves some borderline Br···Br contacts [Br1···Br1 3.8060(11), Br1···Br2 3.8144(7) Å, operators 1–*x*, 2–*y*, 1–*z* and *x*, 1+*y*, *z*, respectively]. Compound **4** is also broadly similar [C3—C4—C17—O1 –9.8(4), C14—C15—C19—O2 145.7(2), C4—C17—C18—Br1 –69.3(3), C15—C19—C20—Br2 –93.2(2)°], and there are three Br···Br contacts, one of them very short [Br1···Br3 3.5519(4), Br1···Br2 3.7308(4), Br1···Br1 3.7383(5); operators 1½–*x*, –½+*y*, *z*; 1–*x*, 1–*y*, 1–*z*; 1–*x*, 2–*y*, 1–*z*, respectively].

2. Elemental analysis

Elemental analyses (C, H) were conducted using a CE440 Elemental Analyser; the results were found to be in good agreement (±0.3%) with the calculated values.

Table 2. Elemental analysis data for compounds **2**–**4** and **6**–**8**.

Compound	% C		% H	
	calcd.	found	calcd.	found
**2**	82.16	82.40	6.89	6.91
**3**	53.36	53.46	4.03	4.04
**4**	45.40	45.35	3.24	3.23
**6**	58.83	58.94	5.70	5.71
**7**	44.89	44.80	4.06	4.05
**8**	55.89	55.83	3.41	3.40

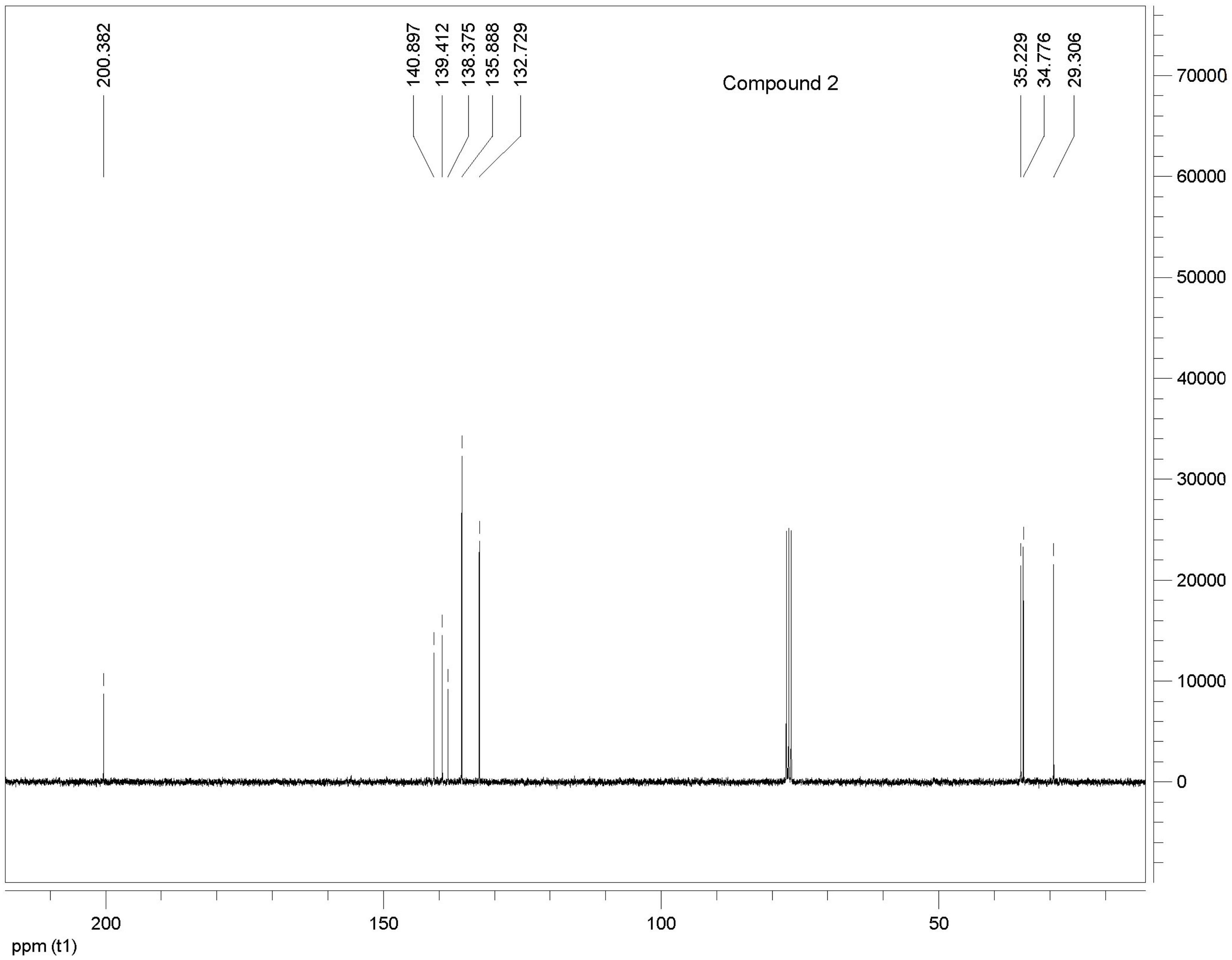
200.382
140.897
139.412
138.375
135.888
132.729
Compound 2
35.229
34.776
29.306
70000
60000
50000
40000
30000
20000
10000
0
200
150
100
50
ppm (t1)

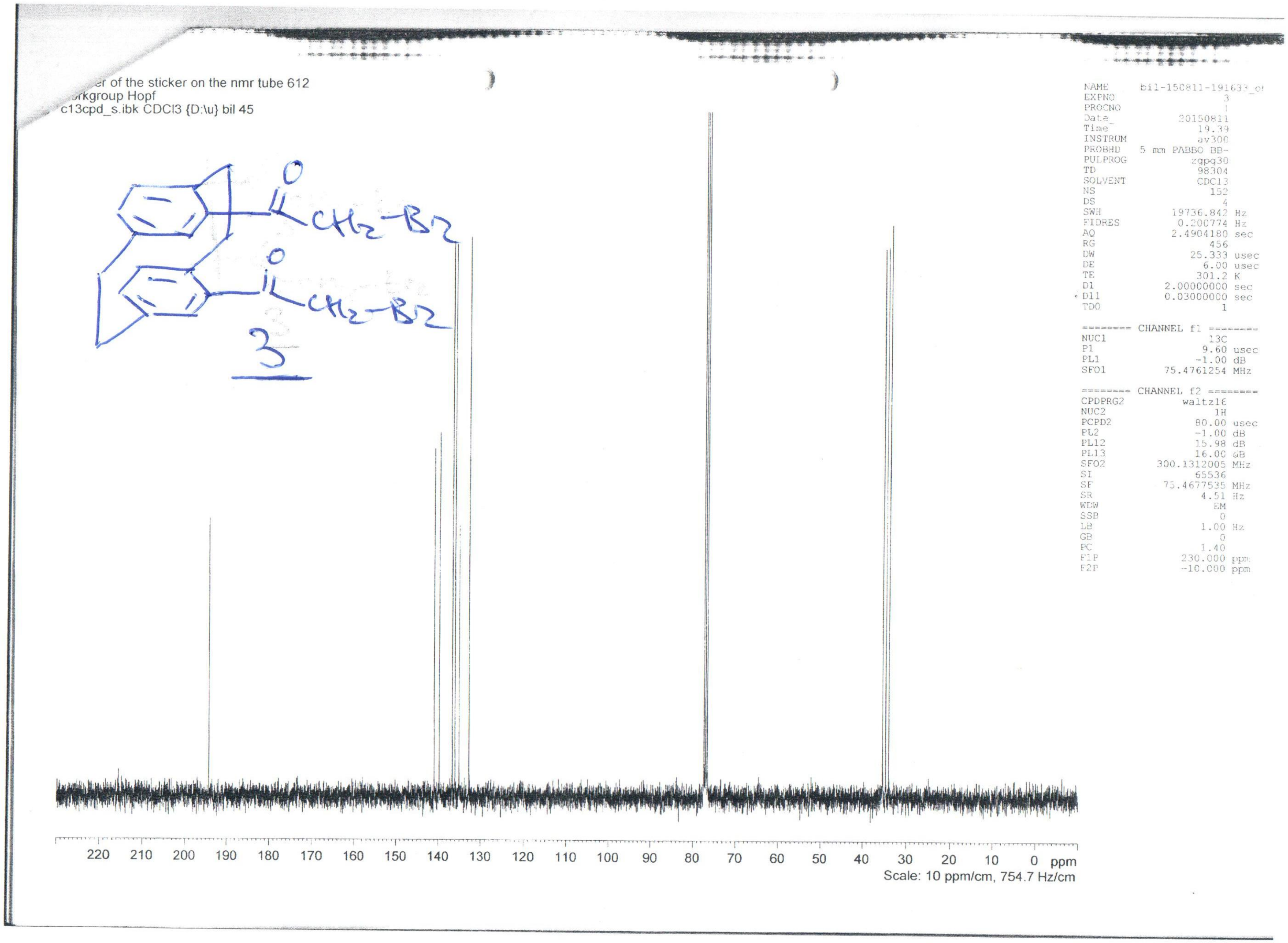
er of the sticker on the nmr tube 612
orkgroup Hopf
c13cpd_s.ibk CDCl3 {D:\u} bil 45
O
CH2-Br
O
CH2-Br
3
NAME bil-150811-191633_o
EXPNO 3
PROCNO 1
Date_ 20150811
Time 19.39
INSTRUM av300
PROBHD 5 mm PABBO BB-
PULPROG zgpg30
TD 98304
SOLVENT CDCl3
NS 152
DS 4
SWH 19736.842 Hz
FIDRES 0.200774 Hz
AQ 2.4904180 sec
RG 456
DW 25.333 usec
DE 6.00 usec
TE 301.2 K
D1 2.00000000 sec
D11 0.03000000 sec
TD0 1
======== CHANNEL f1 ========
NUC1 13C
P1 9.60 usec
PL1 -1.00 dB
SFO1 75.4761254 MHz
======== CHANNEL f2 ========
CPDPRG2 waltz16
NUC2 1H
PCPD2 80.00 usec
PL2 -1.00 dB
PL12 15.98 dB
PL13 16.00 dB
SFO2 300.1312005 MHz
SI 65536
SF 75.4677535 MHz
SR 4.51 Hz
WDW EM
SSB 0
LB 1.00 Hz
GB 0
PC 1.40
F1P 230.000 ppm
F2P -10.000 ppm
220 210 200 190 180 170 160 150 140 130 120 110 100 90 80 70 60 50 40 30 20 10 0 ppm
Scale: 10 ppm/cm, 754.7 Hz/cm

S5

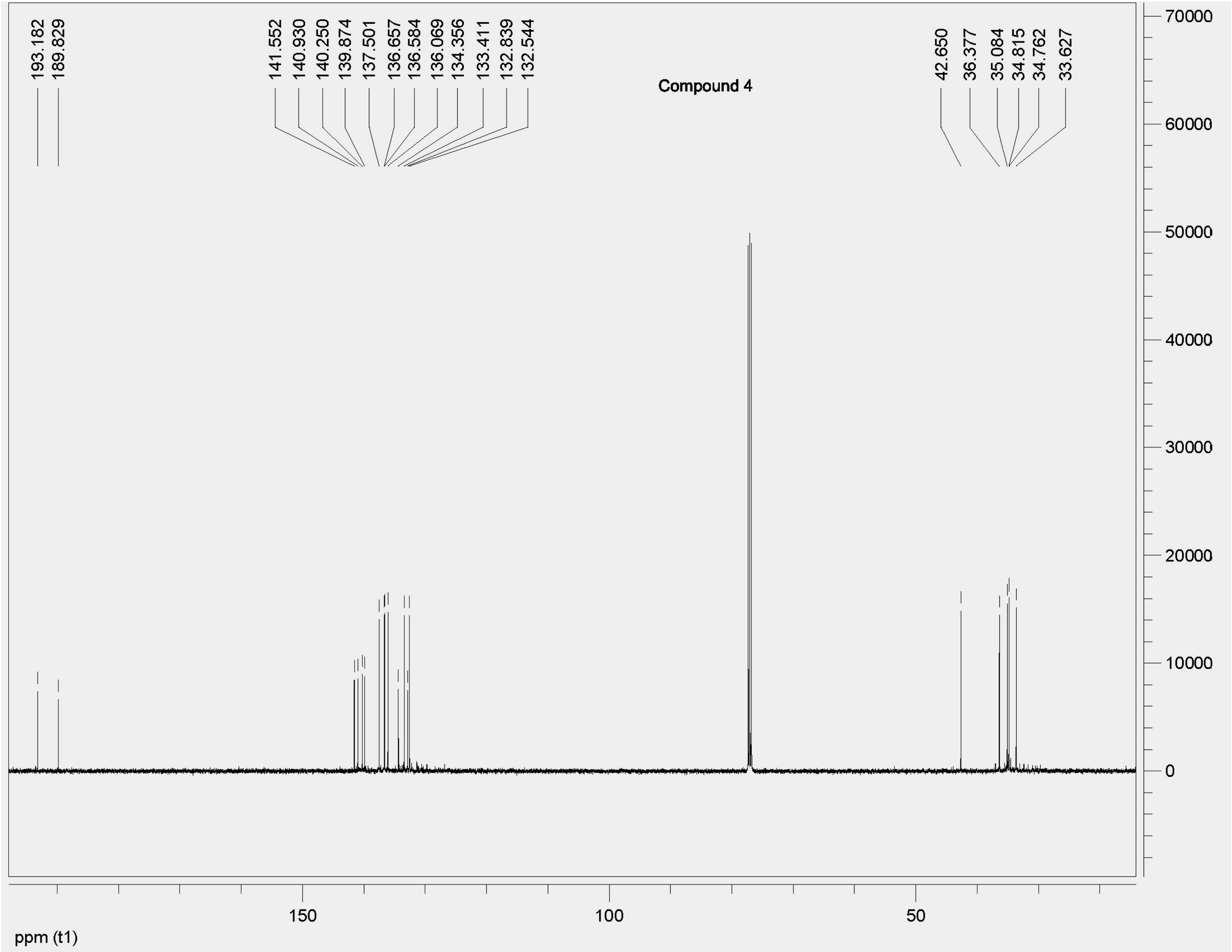
193.182
189.829
141.552
140.930
140.250
139.874
137.501
136.657
136.584
136.069
134.356
133.411
132.839
132.544
Compound 4
42.650
36.377
35.084
34.815
34.762
33.627
70000
60000
50000
40000
30000
20000
10000
0
150
100
50
ppm (t1)

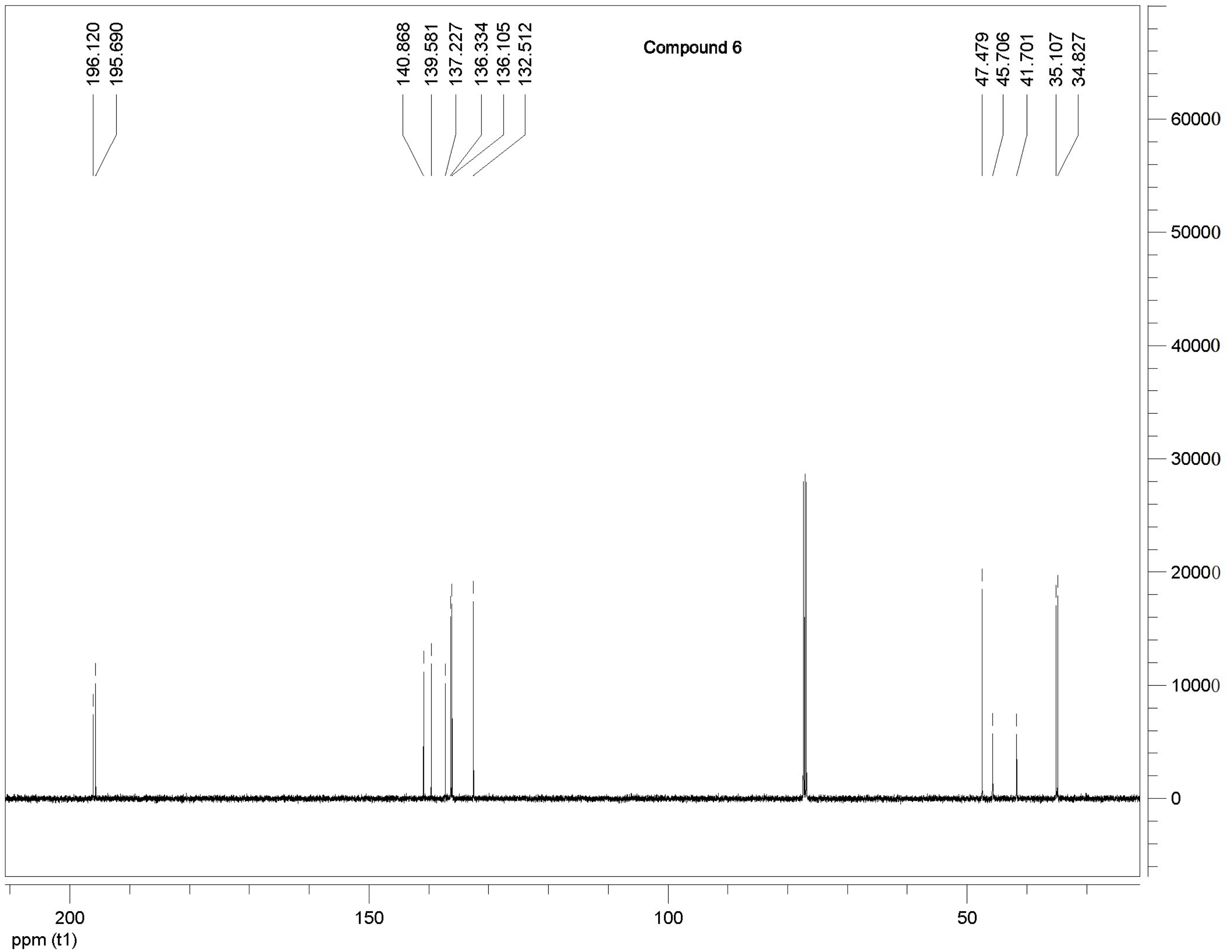

Compound 6
196.120
195.690
140.868
139.581
137.227
136.334
136.105
132.512
47.479
45.706
41.701
35.107
34.827
60000
50000
40000
30000
20000
10000
0
200
150
100
50
ppm (t1)

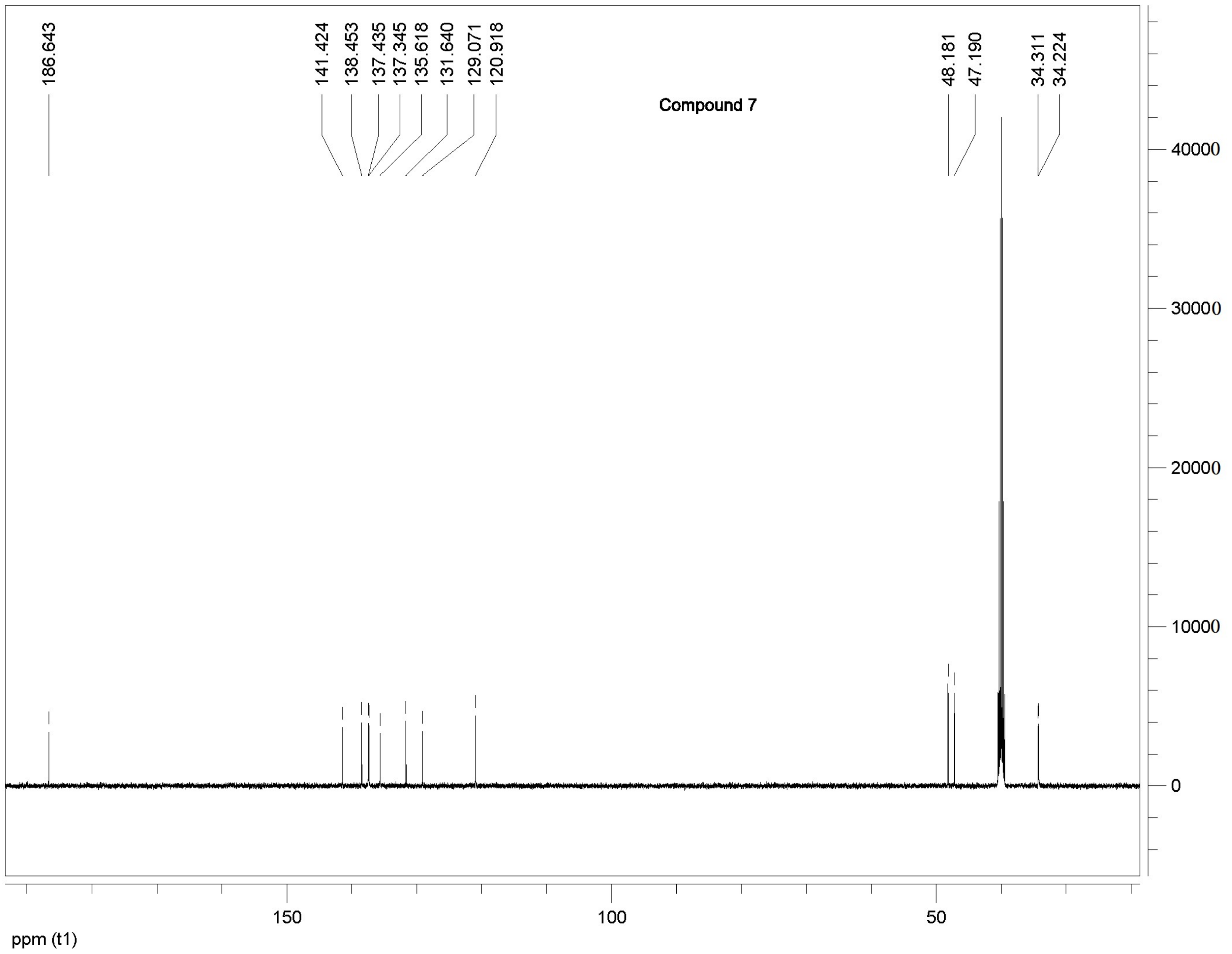
186.643
141.424
138.453
137.435
137.345
135.618
131.640
129.071
120.918
48.181
47.190
34.311
34.224
Compound 7
40000
30000
20000
10000
0
150
100
50
ppm (t1)

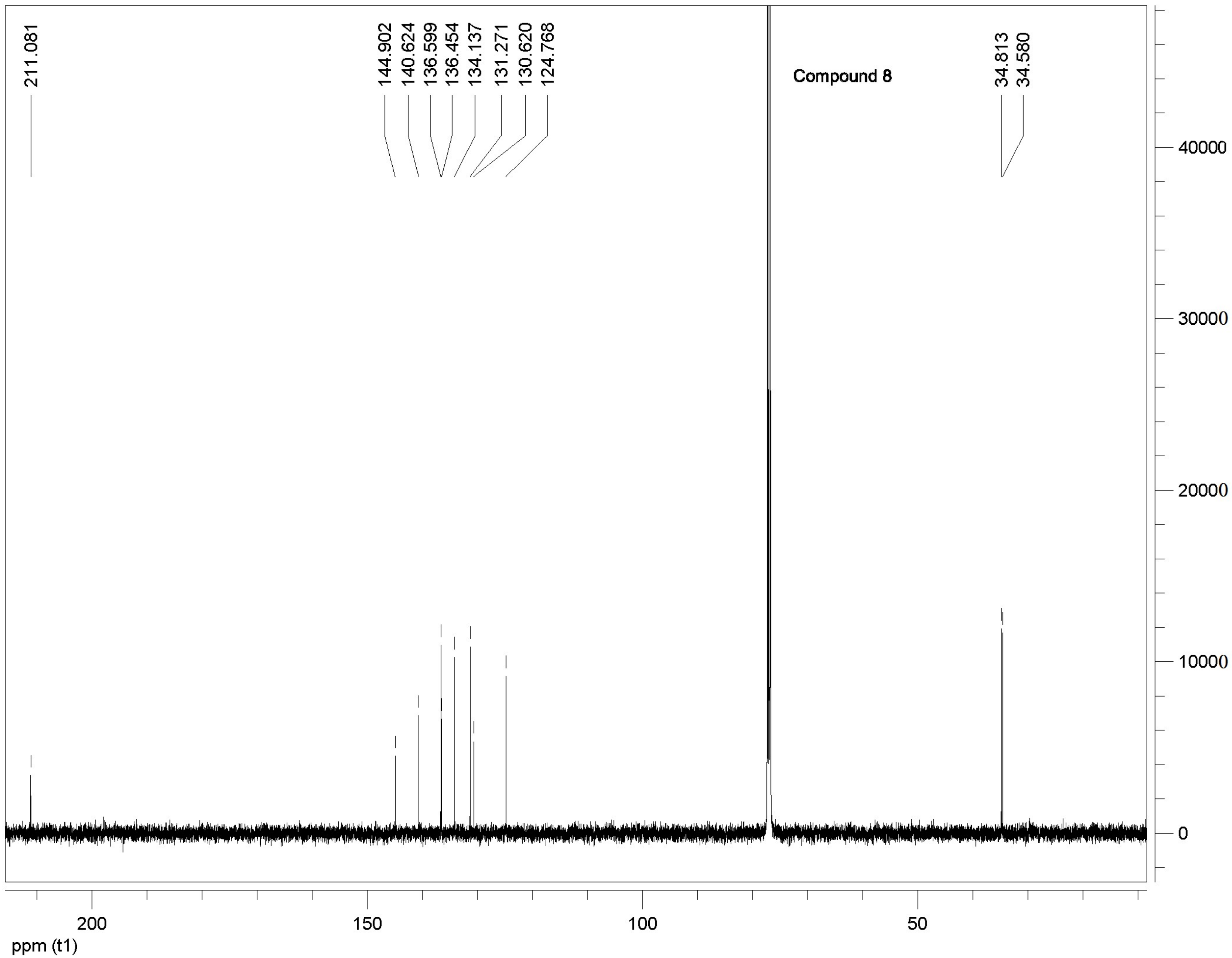
211.081
144.902
140.624
136.599
136.454
134.137
131.271
130.620
124.768
Compound 8
34.813
34.580
40000
30000
20000
10000
0
200
150
100
50
ppm (t1)
